# Rates of Alexithymia and Its Association With Smartphone Addiction Among a Sample of University Students in Egypt

**DOI:** 10.3389/fpsyt.2020.00304

**Published:** 2020-04-24

**Authors:** Hussien Elkholy, Mahmoud Elhabiby, Islam Ibrahim

**Affiliations:** Department of Neurology and Psychiatry, Faculty of Medicine, Ain Shams University, Cairo, Egypt

**Keywords:** alexithymia, smartphone addiction, university students, behavioral addiction, Smartphone Addiction Scale–Short Version (SAS-SV), Toronto Alexithymia Scale (TAS)

## Abstract

**Introduction:**

Alexithymia is characterized by difficulties in describing feelings. Many studies have shown that there is a relation between alexithymia and different types of addictions. Nowadays, smartphone addiction is proposed to be a global problem. The current study focuses on the rates of alexithymia and its association with smartphone addiction in an Egyptian university.

**Materials and Methods:**

This is a cross-sectional study that was conducted in Ain Shams University. A sample of 200 university students was surveyed using Toronto Alexithymia Scale (TAS) and Smartphone Addiction Scale***–***Short Version (SAS-SV).

**Results:**

The results showed that 44 students (22%) had alexithymia. It was also found that around one third of the sample (N=65, 32.5%) met the criteria of smartphone addiction. There was a strong association between alexithymia and smartphone addiction (OR=4.33, 95% CI 2.15–8.74, p= < 0.001).

**Conclusion:**

This study supports existing literature indicating the strong association between alexithymia and smartphone addiction.

## Introduction

Alexithymia is characterized by difficulties describing feelings and a decreased ability to differentiate between emotions and physical sensations (1). It was first studied in patients with psychosomatic disorders (2). Those suffering from alexithymia are unable to verbalize their own emotions and/or the emotions of others. Alexithymia has been linked to difficulties in facing and dealing with stressful conditions (3), anxiety and depression (4), lower self-esteem, dissociative experiences (5), low relationship satisfaction (6), and difficulties in building and maintaining healthy interpersonal relationships (7).

Moreover, many studies have shown that there is a relation between alexithymia and different types of addictions; including opioid use disorder (8), alcohol use disorder (9), and behavioral addictions like internet addiction (10).

On the other hand, smartphones are devices capable of processing information that include access to Internet and social networks, messaging, and multimedia besides their main function as a tool for communication (11). As individuals almost always have their smartphones with them, and they can use their smartphones multiple times during the day, smartphone use may become an automatic behavior that is performed with little thinking (12). Therefore, a great concern has emerged that high frequency smartphone use may suggest that smartphones could become a behavioral addiction. However, the fifth version of the Diagnostic and Statistical Manual of Mental Disorders (DSM-5) does not recognize smartphone addiction as a disorder (13).

Individuals with symptoms of smartphone addiction tend to bring their phone with them wherever they are and think about their phone even if they cannot use it, which ultimately influences daily tasks. The proposed criteria to determine whether an individual suffer from smartphone dependence consists of four main components: compulsive phone use, tolerance witnessed by longer and more intense use, withdrawal symptoms, and functional impairment by interfering with other life activities (14).

Despite the advantages of providing information and communication opportunities (15) and not yet being officially recognized as a disorder (13), smartphone overuse or addiction is proposed to have negative impacts on health. Physical health problems such as visual impairment, musculoskeletal problems (16, 17), ear pain, headache, and sleep disorders, (18, 19) have been suggested to be related to it. Moreover, some mental health problems have also been linked to smartphones addiction such as depression and anxiety (20).

On the other hand, as individuals with alexithymia complain of having difficulties in identifying and describing their feelings, thus, they may resort to internet use to manage them (21). With the rapid development of smartphones, internet use became easier and more accessible (22). Thus, the use of smartphone and even dependence on it might be higher among this group. Few previous studies have investigated the relation between alexithymia and smartphone addiction and the results were interesting. Alexithymia was positively correlated with mental health problems and smartphone addiction (23–25). Yet, to our knowledge, no studies have explored such relation in Arab countries.

Furthermore, many smartphone addiction studies were focused on college students. They may spend much time on “interaction” with the mobile phone which may affect their daily life. Moreover, researchers suggest that the higher the degree of mobile phone addiction, the greater the likelihood that college students’ academic performance will be affected (26). Hence, the current study focuses on alexithymia and its relation to smartphone addiction in students at an Egyptian university.

## Materials and Methods

This is a cross-sectional study that was conducted in Ain Shams University, which is in Cairo, Egypt, from April to August 2019. The study obtained the approval of the Research Ethics Committee (REC) of Faculty of Medicine (approval no. FMASU: R 45/2019). The sample size was calculated to be 200 students setting alpha error at 5% and confidence interval width at 0.1 (margin of error at 5%). Prevalence of alexithymia among university students was shown to be 12.5% in a previous study (27). The sample size was calculated taking in account 10% drop out rate. Students were recruited from campus and asked to complete a questionnaire including some demographic data and two scales. A total of 200 students participated in the study. The participants were requested to fill in a traditional paper-and-pencil questionnaire. They were informed about the aim of the study and that by filling in the questionnaire they consent to take part. It took the respondents approximately 15 min to complete the questionnaire.

### Measures

Demographic information collected includes age, sex, faculty, and academic year. The questionnaire included two scales: Toronto Alexithymia Scale and Smartphone Addiction Scale–Short Version.

#### Toronto Alexithymia Scale (TAS-20), Arabic Version

Toronto Alexithymia Scale, (28) Arabic Version (29) was used to measure alexithymia. TAS is the most used and extensively validated measure for alexithymia (30). TAS consists of 20 items that is divided into three subscales: difficulty identifying feelings (DIF) with seven items, difficulty describing feelings (DDF) with five items, and externally oriented thinking (EOT) with eight items. TAS is five-point Likert scale ranging from 1 “strongly disagree” to 5 “strongly agree,” with five items negatively keyed. The total score is calculated by summing the responses of all the items with a range of 20–100. The cutoff point is 60 and people with score of more than 60 are considered as having alexithymia. People with scores between 52 and 60 are considered to have possible alexithymia. People with a score of 51 or below are considered free of alexithymia. This tool has been translated to Arabic and back translated to the original language by a panel of experts following the World Health Organization (WHO) guidelines for translation of instruments. The scale showed good internal consistency reliability, test–retest reliability, convergent, discriminant, and concurrent validity. In this study, we arranged the sample into two groups using the score of 60 as a cutoff point for having alexithymia or not.

#### The Smartphone Addiction Scale–Short Version (SAS-SV), Arabic Version

The SAS-SV is a validated scale which was originally designed in South Korea but published in English (31, 32). This scale is a shortened version of the original 40 itemed scale. It is a ten itemed questionnaire used to assess levels of smartphone addiction. Participants are asked to rate on a dimensional scale how much each statement relates to them. The total score ranges from 10 to 60. This scale has been used in various research across different cultures including (33, 34). The scale is very quick and easy to use, there are no reverse scores involved. Smartphone addiction cut-off values of ≥ 31 and ≥ 33 for male and female participants, respectively, were applied as suggested by Kwon et al. (31, 32). Arabic version of SAS-SV used in this study was translated and validated by Sfendla et al. (35) and showed excellent reliability based on Cronbach’s alpha (α = 0.94).

### Data Analysis

The SPSS 22.0 was used in our study for statistical analyses. The significance level was set at 0.05. Chi square and T-test were used to check the differences regarding alexithymia and smartphone addiction among the demographic features. Odds ratio was used to express the magnitude of the association between alexithymia and smartphone addiction.

## Results

### Demographic Features of the Participants

Data were collected from 85 males (42.5%) and 115 females (57.5%). The age of the participants ranged from 17 to 27 years with a mean age of 21.225 ± 1.986. Eighty-four students (42%) were enrolled in practical faculties (i.e. medicine, engineering, etc.) and 116 (58%) students were enrolled in theoretical faculties (i.e. law, commerce, etc.). Regarding academic years, 21 students (10.5%) were enrolled in the first year, 38 students (19%) were enrolled in second year, 48 students were enrolled in third year (24%), 72 students (36%) were enrolled in fourth year, 17 students (8.5%) were enrolled in fifth year, and only 4 students (2%) were enrolled in sixth year.

### Alexithymia According to TAS-20 Scores

Alexithymia was measured by TAS-20. In this study the cutoff score of 60 was used to differentiate those with and without alexithymia. The results showed that 44 students (22%) had alexithymia, while the remaining 78% were considered negative for it. There was no significant difference between males and females (p=0.917), between practical and theoretical faculties (p=0.391), nor academic year (p= 0.520) regarding alexithymia rates as shown in **Table 1**. There were also no significant differences between males and females (p=0.790), type of faculties (p=0.795), nor different academic years regarding (p=0.750) the scores of TAS as shown in **Table 2**.

**Table 1:** Comparing rates of smartphone addiction and alexithymia according to sex and type of faculty.

		Sex																		Chi square test	
		Female									Male										
		N			%						N			%						p value	sig.
Smartphone Addiction (SAS-SV)	Negative	72			62.6%						63			74.1%						0.086	NS
	Positive	43			37.4%						22			25.9%							
Alexithymia (TAS)	No Alexithymia	90			78.3%						66			77.6%						0.917	NS
	Alexithymia	25			21.7%						19			22.4%							
		**Faculty**																		**Chi square test**	
		**Practical**									**Theoretical**										
		**N**			**%**						**N**			**%**						**p value**	**Sig**.
Smartphone Addiction (SAS-SV)	Negative	58			69.0%						77			66.4%						0.691	NS
	Positive	26			31.0%						39			33.6%							
Alexithymia (TAS)	No Alexithymia	68			81.0%						88			75.9%						0.391	NS
	Alexithymia	16			19.0%						28			24.1%							
		**Academic Year**																		**Chi Square test**	
		**One**			**Two**			**Three**			**Four**			**Five**			**Six**				
		**N**	**%**		**N**	**%**		**N**	**%**		**N**	**%**		**N**	**%**		**N**	**%**		**p value**	**Sig**.
Smartphone Addiction (SAS-SV)	Negative	17	81.0%		27	71.1%		32	66.7%		44	61.1%		12	70.6%		3	75.0%		0.626	NS
	Positive	4	19.0%		11	28.9%		16	33.3%		28	38.9%		5	29.4%		1	25.0%			
Alexithymia (TAS)	No Alexithymia	15	71.4%		33	86.8%		36	75.0%		54	75.0%		14	82.4%		4	100%		0.520	NS
	Alexithymia	6	28.6%		5	13.2%		12	25.0%		18	25.0%		3	17.6%		0	0.0%			

SAS-SV, Smartphone Addiction Scale–Short Version; TAS, Toronto Alexithymia Scale.

**Table 2:** Relation between scores of SAS-SV and TAS and different factors.

		Sex							T Test	
		Females (N=115)			Males (N=85)				P value	Sig.
SAS-SV	Mean ± SD	31.504 ± 11.716			26.871 ± 9.723				0.003	S
TAS	Mean ± SD	55.948 ± 14.063			56.565 ± 18.694				0.790	NS
		**Faculty**							**T test**	
		**Practical** **(N=84)**			**tdeoretical** **(N=116)**				**P value**	**Sig.**
SAS-SV	Mean ± SD	28.560 ± 11.555			30.241 ± 10.802				0.293	NS
TAS	Mean ± SD	56.560 ± 17.831			55.957 ± 14.894				0.795	NS
		**Academic Year**							**ANOVA**	
		**One** **(N=21)**	**Two** **(N=38)**	**Three** **(N=48)**	**Four** **(N=72)**	**Five** **(N=17)**	**Six** **(N=4)**		**P value**	**Sig.**
SAS-SV	Mean ± SD	23.381 ± 8.891	29.632 ± 12.314	29.042 ± 10.244	31.361 ± 11.493	30.529 ± 9.049	29.750 ± 15.945		0.125	NS
TAS	Mean ± SD	61.048 ± 30.556	54.316 ± 11.414	55.292 ± 14.265	56.667 ± 14.900	55.059 ± 12.060	56.500 ± 4.726		0.750	NS

SAS-SV, Smartphone Addiction Scale–Short Version; TAS, Toronto Alexithymia Scale.

### Smartphone Addiction According to SAS-SV Scores

Smartphone addiction was measured by SAS-SV scale and results showed that around one third of the sample (N=65, 32.5%) met the criteria of smartphone addiction. There was no significant difference between males and females (p=0.086) nor practical and theoretical faculties (p=0.691) regarding smartphone addiction rate as shown in **Table 1**. No significant differences in SAS-SV scores were found between faculties (p=0.293) nor different academic years (p=0.125), however, females had significantly higher scores on SAS-SV (p=0.003) as shown in **Table 2**.

### Relation Between Alexithymia and Smartphone Addiction

There was a strong association between alexithymia and smartphone addiction (OR=4.33, 95% CI: 2.15–8.74, p= < 0.001) as shown in **Table 3**. Also, logistic regression analysis for predictors of smartphone addiction only showed presence of alexithymia as a significant factor as shown in **Table 4**.

**Table 3:** Relation between alexithymia and smartphone addiction.

			Smartphone addiction (SAS-SV)		OR (95% CI)	P value	Sig.
			Negative	Positive			
Alexithymia (TAS)	No alexithymia	N	117	39	4.33 (2.15–8.74)	<0.001	S
		% of TAS	75.0%	25.0%			
		% of SAS	86.7%	60.0%			
	Alexithymia	N	18	26			
		% of TAS	40.9%	59.1%			
		% of SAS	13.3%	40.0%			

SAS-SV, Smartphone Addiction Scale–Short Version; TAS, Toronto Alexithymia Scale.

**Table 4:** Logistic regression analysis for predictors of smartphone addiction.

Dependent SAS-SV	OR	95% C.I. for OR	p value	Sig.
Sex (ref. Male)	1.85	0.96–3.58	0.067	NS
Faculty (ref. Theoretical)	1.04	0.55–1.99	0.898	NS
Age	1.11	0.95–1.3	0.197	NS
TAS (ref. negative)	4.72	2.29–9.73	<0.001	S

SAS-SV, Smartphone Addiction Scale–Short Version; TAS, Toronto Alexithymia Scale.

## Discussion

The term alexithymia was introduced by Peter Sifneos in 1972 (1) and was originally described in patients with psychosomatic complaints (36). The present study addressed the rates of alexithymia and its relation with smartphone addiction among a sample of university students. To our knowledge this is the first study to address the topic in Arab countries and one of the few worldwide.

This study showed that the rate of alexithymia for the whole sample was 22%, and almost the same in both sexes (21.7% of males and 22.4% of females). This rate is comparable to alexithymia rate (24.6%) among Jordanian university students (37). However, this rate is higher than rates found in western countries; 16.7% among Italian high school students (21) and 17.92% among British undergraduate students (38). This discrepancy may be explained by the cultural belief in Arab countries that emotional expression is a sign of personal weakness (39). Another explanation might be that previous research has found psychosomatic complaints to be higher in Arabic cultures (40).

On the other hand, smartphone addiction is a global problem with multiple complications that have been addressed in multiple research. This study showed smartphone addiction to be present among 32.5% of the sample. Moreover, despite being a not statistically significant difference, rate among females (37.45) was higher than males (25.9%). This rate is comparable to those found in some other studies as 28.7% in the Netherlands (41) and 25% in the United States (42). However other studies reported lesser rates as low as 6% in Italy (43) and 18.8% in Japan (44). On the other hand, the non-statistically significant gender difference of smartphone addiction is consistent with previous studies (44, 45). Yet, some studies have reported that female participants indeed have higher rates of smartphone addiction than males (46, 47).

Regarding the relationship between alexithymia and smartphone addiction, our study results showed that there is a strong association between both. This was consistent with most of previous literature (23, 24). One possible explanation of such relation is that people with alexithymia tend to regulate their emotions by various types of addictive behaviors (48). However, one study showed that individuals with alexithymia had less frequent mobile phone use (49)

Several limitations are present in this study. The cross-sectional study design could not provide the causal relationship between alexithymia and smartphone addiction so longitudinal studies are needed to address this problem. Secondly the study is based on a self-reported questionnaire, which may result in several confounding factors. Thus, a more objective data collection methods should be used to improve credibility in the future. Finally, the small sample size and the collection of data from one university could affect the generalizability of the findings. Despite that the sample size was calculated to be enough, we would recommend larger samples and recruitments from more than one university in future studies.

## Data Availability Statement

The raw data supporting the conclusions of this article will be made available by the authors, without undue reservation, to any qualified researcher.

## Ethics Statement

The studies involving human participants were reviewed and approved by Research Ethics Committee (REC) of Ain Shams University Faculty of Medicine (approval no. FMASU: R 45/2019). Written informed consent from the participants’ legal guardian/next of kin was not required to participate in this study in accordance with the national legislation and the institutional requirements.

## Author Contributions

All authors contributed to the conceptualization and design of the study. II was responsible for the field work. All authors were responsible for data revision. HE and II drafted the first version. HE and ME critically revised and finalized it for publication. All authors approved and contributed to the final version.

## Conflict of Interest

The authors declare that the research was conducted in the absence of any commercial or financial relationships that could be construed as a potential conflict of interest.
